# Thalamic and Visual Network Dysfunction Relates to Tremor Response in Thalamic Deep Brain Stimulation

**DOI:** 10.5334/tohm.1039

**Published:** 2025-08-01

**Authors:** Aimee E. Morris, Babatunde Adeyemo, Meghan C. Campbell, Abraham Z. Snyder, Joel S. Perlmutter, Jonathan W. Mink, Scott A. Norris

**Affiliations:** 1 University of Rochester School of Medicine and Dentistry, Rochester, NY, USA; 2 Department of Neurology, Washington University School of Medicine, St. Louis, MO, USA; 3 Department of Radiology, Washington University School of Medicine, St. Louis, MO, USA; 4 Department of Anatomy and Neurobiology, Washington University School of Medicine, St. Louis, MO, USA; 5 Department of Physical Therapy, Washington University School of Medicine, St. Louis, MO, USA; 6 Pittsford, NY, USA

**Keywords:** Essential tremor, functional connectivity, resting-state fMRI

## Abstract

**Background:**

Essential tremor (ET) is the most common movement disorder in adults, but its pathogenesis is incompletely understood. Deep brain stimulation of the ventral intermediate thalamic nucleus (VIM DBS) provides effective treatment for medically-refractory cases. We aimed to determine how pre-surgical resting-state functional connectivity (FC) in medically-refractory ET relates to VIM DBS clinical response.

**Methods:**

We analyzed resting-state FC MRI in 21 participants with medically-refractory ET who subsequently underwent VIM DBS and 34 matched controls. We applied rigorous quality assurance to minimize motion artifact. Whole-brain correlation matrices were computed across 300 cortical, subcortical, and cerebellar regions and compared across groups using object-oriented data analysis, a powerful novel approach. We used multiple linear regression to determine whether network FC (calculated as mean cross-correlation between nodes) in defined networks predicts VIM DBS response. We assessed regional FC using a seed in motor thalamus.

**Results:**

Whole-brain correlation matrices and regional motor thalamus FC differed significantly between groups. Post-hoc network-level testing revealed decreased thalamus-somatomotor, thalamus-visual, and auditory-visual FC in ET versus controls. Regional FC showed increased primary motor cortex and decreased occipital-parietal and cerebellar FC with motor thalamus in ET relative to controls. Visual-lateral somatomotor network FC negatively predicted tremor improvement with VIM DBS.

**Discussion:**

Whole-brain, network, and regional FC results demonstrate cerebello-thalamo-motor pathway dysfunction in ET. Robust FC differences in motor and visual regions related to VIM DBS outcomes. These results, employing rigorous quality control, support the need for additional investigation into the role of visual cortical networks in ET and DBS response.

## Introduction

Essential tremor (ET) is characterized by isolated bilateral upper limb action tremor of at least three years duration, with or without tremor in other body regions, and absence of other neurological signs [1]. Although ET is the most common movement disorder, the underlying pathophysiologic network mechanisms relating to effective therapeutic intervention, including deep brain stimulation (DBS) are incompletely understood.

Multiple studies have implicated dysfunction in the cerebello-thalamo-cortical network in the pathogenesis of ET, on the basis of electrophysiologic [2], functional neuroimaging [3,4,5,6,7], and neuropathology [8,9,10] data. Despite our growing knowledge, the precise relations between these identified abnormalities, symptomatology, and treatment response in ET remains poorly understood. Neuroimaging may reveal previously unrecognized brain regions or networks that play a key role in ET pathophysiology or its treatment. In particular, neuroimaging provides a comprehensive and simultaneous examination of the whole brain, facilitating the identification of relevant abnormalities. Indeed, prior functional neuroimaging studies have identified abnormalities in various brain regions outside of the cerebello-thalamo-cortical circuit, including visual cortex [11,12], middle temporal cortex [13], and inferior parietal cortex [7]. However, neuroimaging findings in ET outside of the cerebello-thalamo-cortical motor network have been inconsistent.

Resting-state functional connectivity magnetic resonance imaging (rs-fMRI) is a powerful method to study network-level dysfunction that can avoid confounds of interpretation from differences in motor performance or abnormal movements during task-based MRI studies [14].

VIM DBS effectively reduces tremor in patients with medically refractory ET [14]. Positron emission topography neuroimaging has shown that clinically effective VIM DBS produces a robust blood flow response to stimulation in the ventral thalamus and ipsilateral supplementary motor area (SMA), a cortical target of VIM efferents [15]. Prior work has shown that strong structural and functional connectivity between thalamic DBS stimulation sites and primary sensorimotor cortex, SMA, and cerebellum correlates with improved tremor suppression [16,17]. Outside of the cerebello-thalamo-cortical network, thalamo-visual-motor FC has been identified as a potential preoperative predictor of tremor arrest after VIM thalamotomy [18,19,20]. Seed regions derived from VIM DBS stimulation sites in patients with ET applied to large normative datasets have identified thalamic structural and functional connectivity with V1 and V2 (in addition to S1, M1, cerebellum, and superior temporal gyrus) as potential predictors of thalamic DBS response [21]. However, this observation in a normative population has not been adequately tested in patients with ET undergoing DBS.

We hypothesized that ET and healthy control participants differ in whole brain connectomes and that such differences manifest as somatomotor, thalamus, and cerebellum resting state network (RSN) differences. Furthermore, we hypothesized that pre-operative cerebellum-thalamus-somatomotor FC would predict tremor improvement after VIM DBS. We used rs-fMRI with stringent motion artifact reduction [22] and a combination of graph theoretical [23] and classical analytical techniques [24,25] to identify patterns of aberrant FC in participants with medically refractory ET compared with age- and sex-matched healthy controls.

## Methods

### Participants

Adults with a clinical diagnosis of ET [1] causing functional impairment despite optimized pharmacologic intervention (i.e., medically refractory) and scheduled for unilateral VIM DBS surgery were recruited from the Movement Disorders Center at Washington University (St. Louis, MO) between 2008–2017. Participants with other movement disorders, structural brain abnormalities, or major medical conditions were excluded. All ET patients provided informed consent for pre-operative MRI studies and the Washington University DBS Outcomes Project as approved by the Washington University Institutional Review Board (IRB), in agreement with the Declaration of Helsinki. Participants were instructed to continue to take medications as prescribed.

Healthy control data were obtained from rs-fMRI scans of healthy adults collected using the same imaging sequences and exclusion criteria under data sharing protocols approved by the Washington University IRB and in agreement with the Declaration of Helsinki [26,27] on either of 2 MRI scanners of the same make and model and equipped with identical hardware and software. Healthy control data passing quality-assurance criteria (described below) were matched to the ET group by age, sex, and the proportion of scans conducted on each MRI scanner.

Tremor severity was assessed in participants with ET using the Fahn-Tolosa-Marin Tremor Rating Scale [28] parts A and B (TRS) pre-operatively and at their initial DBS programming visit (approximately one month post-operatively) in both the stimulation OFF state and again in the ON state after turning on the stimulators and completing initial programming. Response to DBS (ΔTRS_OFF-ON_) was measured as the absolute difference between OFF and ON TRS scores for the side contralateral to VIM DBS. Although DBS benefit is incomplete immediately after initial programming, we adopted the current study design as medication changes confound long-term interpretation of DBS response and ON/OFF data were not consistently obtained after initial programming in our population.

### Data Acquisition

All participants underwent fMRI scans in a 3 Tesla TRIO scanner (Siemens AG) equipped with a standard 12-channel head coil. Participants completed up to a total of 22 minutes of resting state fMRI over 3 consecutive runs using a gradient echo pulse sequence [200 volumes/run, TR = 2200 ms, TE = 27 ms, 48 axial slices, FOV = 256 mm, flip angle = 90°, 4 mm^3^ isotropic voxels]. Participants were instructed to close their eyes, remain still, and not fall asleep. Participants were directly observed for movement or signs of sleep during scanning and participants were asked about wakefulness at the completion of each run. Any runs during which participants were asleep or had sustained tremor were excluded. Anatomical imaging included one sagittal T1-weighted magnetization-prepared rapid gradient echo (MP-RAGE) scan (T1W) [TE = 3.14 ms, TR = 2400 ms, TI = 1000 ms, flip angle = 8˚, 0.9 mm^3^ isotropic voxels] and one T2-weighted 3D scan (T2W) [TE = 461 ms, TR = 3200 ms, 0.9 mm^3^ isotropic voxels].

### Preprocessing

rs-fMRI data were preprocessed using standard techniques [29]: 1) atlas transformation via composition of affine transforms involving a sequence of co-registrations between the fMRI volumes, T2W and T1W structural images, and Talairach atlas target space; 2) realignment of images to correct for head motion; 3) regression of the voxel-wise mean signal and linear trend; 4) regression of several nuisance variables (motion regression derived by Volterra expansion [30] and signal from: ventricles, white matter and averaged over the whole brain); 5) generation of temporal masks flagging frames contaminated by excess motion (see *Motion Correction and Quality Assurance* below); 6) repeat removal of the voxel-wise mean signal and linear trend and regression of nuisance variables ignoring frames previously censored for motion; 7) linear interpolation to replace censored frames using least-squares spectral estimation (only used for band-pass filtering); 8) temporal band-pass filtering (0.009 Hz < f < 0.08 Hz) of the continuous, interpolated data; and 9) spatial smoothing (6 mm FWHM Gaussian blur in each cardinal direction).

To minimize group-specific atlas registration bias, we generated an atlas representative template from study-derived MP-RAGE data from an equal sampling of ET and healthy control participants [31]. Individual participant data were transformed to atlas space by composition of affine transforms involving a sequence of registrations between the fMRI volumes, T2W and T1W structural images, and the atlas template.

Anatomical T1-weighted images were processed using the FreeSurfer 5.0 default recon-all processing pipeline with manual edits. In brief, the FreeSurfer pipeline includes brain extraction, subcortical segmentation, intensity normalization, topology correction, surface deformation following intensity gradients to define boundaries between white matter, gray matter and cerebrospinal fluid, inflation of the surfaces to a sphere, registration to a spherical atlas, and parcellation of the cerebral cortex [32].

### Motion Correction and Quality Assurance

Motion-related artifact is a major methodological and scientific issue in rs-fMRI research [33]. We applied rigorous quality assurance measures to minimize the impact of motion artifacts and movement confounds, including strict criteria for head motion. Framewise displacement was calculated from low-pass filtered [34] (fc = 0.1 Hz) motion parameters; volumes with filtered framewise displacement exceeding 0.1 mm were excluded [35]. After frame censoring, participants with fewer than 120 included fMRI volumes (i.e., 4.4 minutes of low motion data) were excluded. Preprocessing was then repeated using the updated temporal masks to remove volumes contaminated by motion from the outset of preprocessing.

## Statistical Methods

Inter-group comparisons were performed for each baseline measure using an independent-samples *t-*test for continuous or chi-square test for categorical variables.

### Whole Brain Object-Oriented Analyses

We employed an unbiased approach to assess functional connectivity across the entire brain. Each brain was seeded with a set of 300 spherical seeds, including 239 × 10-mm diameter cortical- and 61 × 8-mm diameter subcortical and cerebellar seeds, providing whole-brain coverage [36], as validated and applied to the studies of Parkinson disease and dystonia [26,27]. Seeds in the cerebral cortex, thalamus, and basal ganglia were assigned to 16 resting state networks following a previously published scheme [27]. Time courses were averaged across all included voxels for each seed and Pearson correlations were computed for each seed pair to create individual subject whole-brain correlation matrices. Correlation values were averaged across participants to obtain group-mean correlation matrices. Group difference matrices were derived from subtraction of Fisher-z transformed group-mean data (control minus ET).

We employed a data-driven approach to identify large-scale group-level differences in resting-state functional connectivity using weighted object-oriented data analysis (OODA) [23]. Most functional neuroimaging studies suffer from the so-called *curse of dimensionality* [37] or ‘*small n, large p*’ problem [38,39] due to the large number of predictor variables (voxels) in fMRI and relatively small number of participants. OODA circumvents these challenges by directly comparing whole-brain connectivity matrices as objects [23]. OODA employs the parametric Gibbs distribution model to whole brain correlation matrices. We determined the central tendencies of each group (g*) matrix under the Gibbs distribution for each group, healthy control and ET, and the Euclidean distance between the healthy control- and ET central g* matrices. To assess statistical significance, we generated a distribution of distances by bootstrapping samples from each dataset (N = 1,000) to generate new central g* matrices as described by Gratton et al [27].

We used multidimensional scaling (MDS) to visually represent the dimensional distribution of individual correlation matrices based on the distances (i.e., dissimilarities) between them [23]. Euclidean distances between each pair of correlation matrices were calculated to generate a pairwise distance matrix. The matrix objects are then represented in a dimensional space where the principal components of distance are the primary dimensions. Each dot on the plot represents an individual correlation matrix and the distance between points correlates with their actual distance. The central tendencies of each group (g*) were also represented in the MDS plots. MDS computations were performed using the cmdscale.m function in MATLAB R2018b.

### Post-Hoc Network Analyses

Following determination of significant whole brain matrix-level FC differences, we examined network-level FC between canonical RSNs to identify matrix-level differences. We calculated composite within- and between-network FC by taking the average pairwise correlation between unique network nodes to generate “block-average” network FC matrices [39,40]. We selected six cross-network FC blocks *a priori* for hypothesis testing: thalamus-dorsal somatomotor, thalamus-lateral somatomotor, and thalamus-cerebellum based on previous work implicating these regions in the pathophysiology of ET; thalamus-visual, visual-lateral somatomotor, and visual-auditory were additionally tested based on the prominence of visual network findings in the OODA analysis and work suggesting relevance of visual networks in ET [11,18,19,20,21].

Independent-samples *t*-tests assessed group-level differences in network FC. We corrected for multiple comparisons with a false discovery rate (FDR) of 10% to determine the significance threshold. FDR was selected because it explicitly informs about the maximum percentage of false positive and is a more powerful approach than family-wise type I error correction results [41]. For analyses in which the consequences of a false positive finding are relatively benign, a 5% FDR is too conservative and a 10–20% FDR is considered appropriate [42].

We examined medication effects in network-to-network blocks that significantly differed between ET and healthy control groups. We stratified ET participants by whether they were taking propranolol or primidone, the two most common medications in this cohort and applied a non-parametric Mann-Whitney-U test to determine whether either medication was associated with differences in FC. Other medications could not be assessed as they were taken by fewer than five participants.

### Regression of Network-Level FC with Clinical Measures

To determine rs-fMRI markers of clinical relevance, we calculated a multiple linear regression to predict *a priori* network FC values based on tremor response (ΔTRS_OFF-ON_), medication status for drugs with significant effects on network FC in this study (primidone), and the scanner used for data collection in ET participants. The visual-lateral somatomotor and thalamus-visual networks were chosen based on the combination of our network-level findings and prior reports of thalamo-visual-motor FC as a preoperative predictor of tremor arrest after VIM thalamotomy [18,19,20,21]. The cerebellar-thalamus and thalamus-lateral somatomotor networks were chosen based on literature implicating cerebello-thalamo-motor FC in the pathophysiology of tremor [4,43,44]. Significance thresholds were Bonferroni corrected for multiple comparisons. Significance was defined as p < 0.025.

### Regional Analyses

To further explore the spatial extent of the thalamic motor network in ET, we used a hypothesis-driven, seed-based approach to study motor thalamus FC across the entire brain [4]. We combined the four thalamus seeds assigned to the dorsal somatomotor network [36] into one bilateral motor thalamus seed containing the ventral intermediate nucleus (VIM), the principal cerebellar outflow target and our cohort’s target for deep brain stimulation in essential tremor.

For each participant, time series data were extracted from each voxel within the seed and averaged over all included voxels. White matter and ventricular signals were masked from the seed image. Correlation maps were computed between each seed and all voxels in the brain using the Pearson product moment formula. Maps were transformed using Fisher z-transform to obtain z(r) maps that were averaged over participants to obtain group-mean correlation maps and group difference (control minus ET) z(r) images. To assess statistical significance, random effects analyses (voxel-wise t-map) were done at the group level and t-images were converted to equally probable Z-images and assessed for the presence of significant voxel clusters. Cluster-wise significance criteria were determined by non-parametric analysis of surrogate Z-images generated by Monte-Carlo permutation (10,000 iterations) simulation of the null hypothesis and corrected for multiple comparisons with a fixed false-positive rate of 5% [45].

## Results

### Participants

We recruited 40 participants with ET and identified a like number of sex- and age-matched healthy controls. 13 participants were excluded prior to analysis (2 had comorbid neuropsychiatric diagnoses, 5 had observed rest tremor throughout scanning, and 2 had anatomic abnormalities on structural MRI). After application of rigorous quality assurance criteria, 21 participants with ET and 34 age/sex matched healthy control participants remained. All ET participants had bilateral upper limb tremor and subsequently underwent unilateral VIM DBS targeting the limb in which tremor caused more functional impairment (4 R VIM, 17 L VIM). Electrode placement in the VIM was visually localized and confirmed by post-operative CT scan co-registered to pre-operative MRI. Table 1 provides detailed demographic and clinical information for analyzed ET participants. Groups did not differ in demographic, MRI scanner, or fMRI motion parameters (Table 2).

**Table 1:** Demographic information: participants analyzed with essential tremor.


PARTICIPANT^a^	AGE(YEARS)	SEX	FAMILY HISTORY	DURATION (YEARS)	TRS^b^	ΔTRS_(OFF-ON)_^b^	DOMINANT HAND	TREMOR MEDICATIONS(TOTAL DAILY DOSE IN MG)	INITIAL STIMULATION PARAMETERS				

									SITE	CONTACTS	V	PW	FREQ

ET1	66	F	sporadic	41	22	0	Right	propranolol 120, primidone 500, gabapentin 1800	L VIM	C+2-	1.0 V	60 µs	185 Hz

ET2	80	F	familial	12	44	2.5	Right	propranolol 140, primidone 750, gabapentin 300	L VIM	0–3+	2.3 V	60 µs	185 Hz

ET3	69	M	familial	41	41	8	Right	primidone 500, clonazepam 1.5	L VIM	C+0-	2.6 V	60 µs	185 Hz

ET4	77	M	familial	47	21	6	Right	primidone 350, metoprolol 25	L VIM	0–3+	2.5 V	60 µs	185 Hz

ET5	65	M	sporadic	55	36	2.5	Right	primidone 750	L VIM	C+1-	1.5 V	60 µs	185 Hz

ET6	58	F	sporadic	12	59	5.5	Right	propranolol 40, gabapentin 300	L VIM	C+2-	3.0 V	60 µs	185 Hz

ET7	62	M	familial	47	21	1	Right	propranolol 80	L VIM	C+2-	1.5 V	60 µs	185 Hz

ET8	55	M	familial	30	20	1	Right	clonazepam 3, carbidopa-levodopa 100/400	L VIM	1–3+	2.8 V	60 µs	185 Hz

ET9	48	M	sporadic	18	29	2	Left	propranolol 320, primidone 600	R VIM	1–3+	2.5 V	60 µs	185 Hz

ET10	81	M	familial	41	31	3	Right	propranolol 80	L VIM	1–3+	2.5 V	60 µs	185 Hz

ET11	62	M	sporadic	12	14	1	Right	none	L VIM	C+2-	2.0 V	60 µs	185 Hz

ET12	55	M	sporadic	10	27	5	Right	diazepam 30, zolpidem 10	R VIM	0+3-	2.0 V	60 µs	185 Hz

ET13	56	M	familial	2^c^	23	5	Right	propranolol 200, primidone 750	L VIM	1–3+	2.0 V	60 µs	185 Hz

ET14	52	F	sporadic	38	38	3	Right	carbidopa/levodopa 75/300	L VIM	0–3+	2.0 V	60 µs	185 Hz

ET15	49	F	sporadic	11	20	3.5	Right	clonazepam 1, carbidopa-levodopa 150/600	R VIM	1–3+	1.6 V	60 µs	185 Hz

ET16	66	M	familial	6	15	2.5	Right	none	L VIM	0–3+	1.3 V	60 µs	185 Hz

ET17	75	F	sporadic	35	18	2	Right	topiramate 200, primidone 500	L VIM	0+3-	2.0 V	60 µs	185 Hz

ET18	62	F	familial	10	30	2	Right	topiramate 100, propranolol 160	L VIM	1–3+	2.0 V	60 µs	185 Hz

ET19	76	M	familial	36	26	2	Right	propranolol 180, primidone 400	L VIM	C+1–3+	1.5 V	60 µs	185 Hz

ET20	55	F	familial	52	30	2.5	Left	pramipexole 1	R VIM	C+0+2-	2.0 V	60 µs	185 Hz

ET21	57	M	familial	38	21	2	Right	topiramate 200, primidone 1000	L VIM	C+1-	1.8 V	60 µs	130 Hz


^a^19 additional participants were excluded from final analyses (N = 5: observed movement throughout scanning, N = 2: comorbid neuropsychiatric diagnoses, N = 2: anatomic abnormalities on structural MRI, N = 8: did not meet motion quality assurance criteria, N = 2: to correct group imbalance of the MRI scanners used for data collection). ^b^TRS represents the total score while TRS_Off-On_ reflects only tremor scores for limbs contralateral to VIM surgery. ^c^Clinical follow-up confirmed diagnosis of ET (symptoms >3 years). Abbreviations: V = Voltage, PW = Pulse Width; Freq = Frequency.

**Table 2:** Group matching data: essential tremor and control.


	ET (N = 21)MEAN (SD)	CONTROL (N = 34)MEAN (SD)	STATISTIC	*P* VALUE

Age in years [range]	63.1 (10.1) [48–81]	61.9 (8.0) [49–82]	*T* = 0.5	0.62

Sex	38% F, 62% M	38% F, 62% M	χ^2^ = 0.00	1.00

Scanner	43% S1, 57% S2	41% S1, 59% S2	χ^2^ = 0.02	0.90

Handedness	90% right-handed	88% right-handed	χ^2^ = 0.07	0.80

Root mean square framewise displacement (mm)	0.056 (0.008)	0.053 (0.008)	*T* = 3.34	0.17

Number of frames kept	408.0 (107.8)	446.0 (118.2)	*T* = –1.19	0.24

TRS [range]	27.9 (10.9) [14–59]			


### ET was Characterized by Differences in the Whole-Brain Functional Correlation Matrix

**Figure 1:**
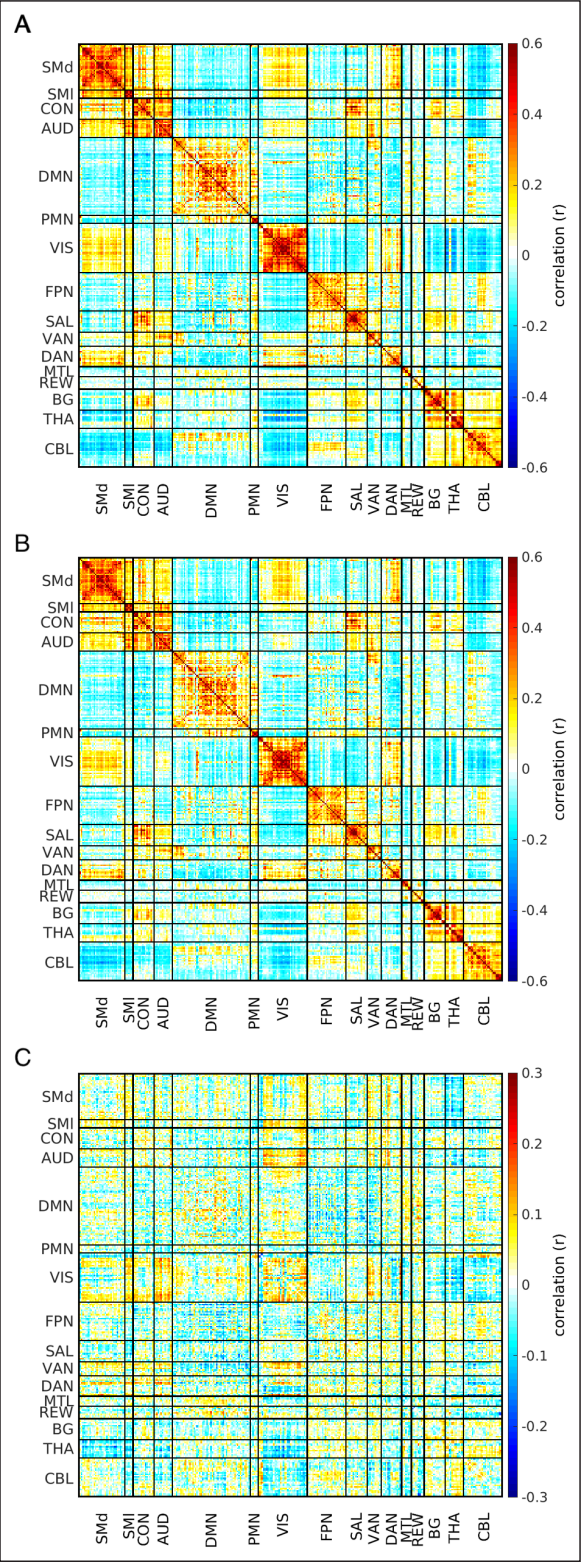
**Group-level connectomes in ET and Controls.** Large-scale functional connectomes in **(A)** Control and **(B)** ET participants reveal grossly similar resting state network organization, consistent with resting state network architecture found in other cohorts of healthy adults. Warm and cool colors indicate positive and negative correlations, respectively. **C:** A subtraction matrix (Control minus ET) shows selective network-to-network blocks of altered FC in ET, particularly involving thalamus and visual internetwork FC.

**Figure 2:**
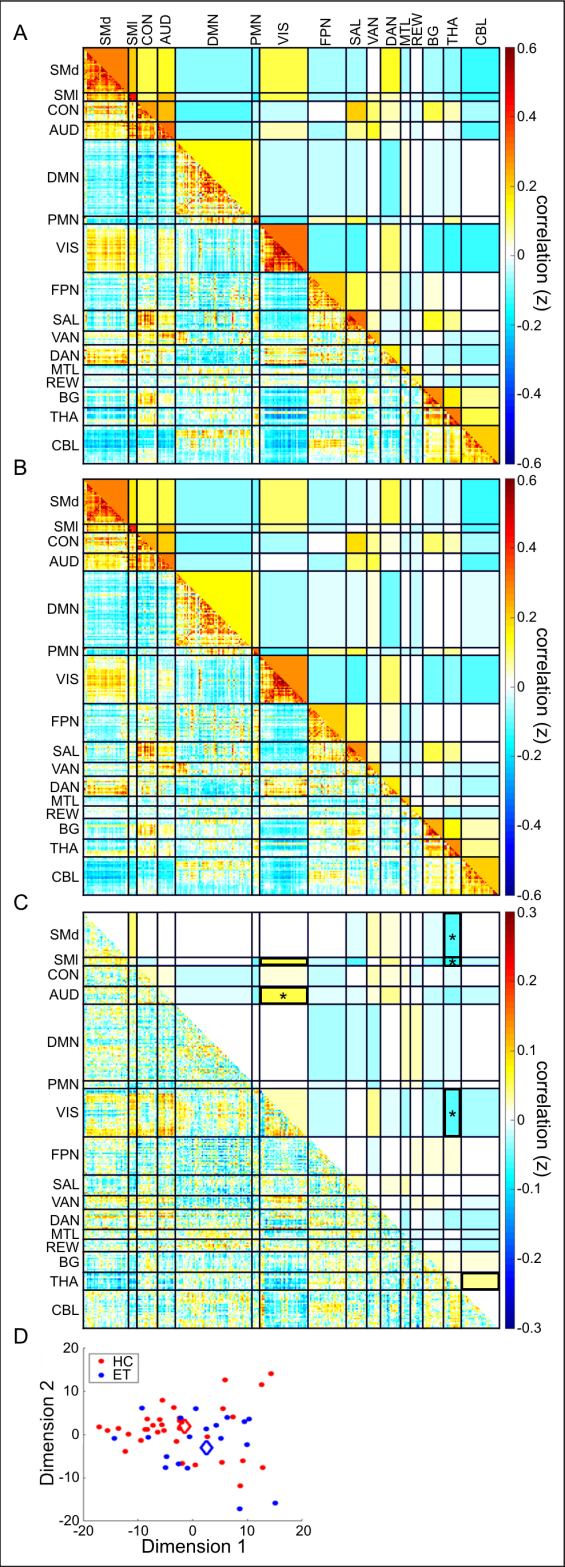
**Disruption of large-scale network structure in ET.** Central weighted connectome object (g*) for **(A)** Control and **(B)** ET groups and **(C)** subtraction (Control g* – ET g*). Upper triangles show composite block FC scores (average cross-correlation between seeds) while the lower triangles show the matrix objects with all edges preserved. Connectome objects differ significantly between ET and controls. Black outlines in the upper triangle indicate blocks chosen a priori for hypothesis testing of ET versus controls. Stars indicate blocks with significantly different network FC between ET and controls. Note the color scale difference in (C). **D:** Multidimensional scaling plot demonstrates separation of ET and control connectomes represented in 2-dimensional space. Diamonds indicate the central object for each group.

Group-average correlation matrices for both the healthy controls and the ET participants showed typical network-related block structure characterized by high within-network FC (on diagonal blocks) and weakly positive or anti-correlated between-network FC (off-diagonal blocks) [36,46] (Figure 1). We computed each group’s central matrix object (g*) (essentially the group average correlation matrix) and their difference (HCl g* - ET g*) (Figure 2). This difference, taken as a whole, was statistically significant (p = 0.048). Post-hoc network level FC analyses revealed statistically significant effects involving the thalamus, somatomotor, visual, and auditory networks. Specifically, participants with ET showed less negative (i.e., weaker) dorsal somatomotor, lateral somatomotor and visual network correlations with the thalamus. Similarly, auditory and inferior somatomotor cortices showed less positive (i.e., weaker) correlations with visual areas (Figure 1C). Network-level FC block-average matrices are illustrated in the upper triangles of Figure 2. The Benjamini-Hochberg-corrected significance threshold (FDR 10%) was *p* < = 0.040.

### Altered Thalamic and Visual-Auditory Network-Level FC in ET

For the six *a priori* inter-network FC blocks, the ET group had significantly different FC between thalamus-lateral somatomotor, thalamus-dorsal somatomotor, thalamus-visual, and visual-auditory networks compared with healthy controls (Table 3). ET and healthy control groups did not differ in visual-lateral somatomotor or thalamus-cerebellum FC.

**Table 3:** ET versus Health Control (HC) *a priori* network- (top) and regional- (bottom) functional connectivity (FC).


*A PRIORI* NETWORK	CONTROLS	ET	t-SCORE	p

Thalamus – Lateral Somatomotor	–0.07	0.00	2.89	**< 0.01** ^a^

Thalamus – Dorsal Somatomotor	–0.08	–0.03	2.50	**0.02** ^a^

Thalamus – Visual	–0.13	–0.08	2.17	**< 0.04** ^a^

Thalamus – Cerebellum	0.11	0.08	–1.70	0.09

Visual – Lateral Somatomotor	0.12	0.07	–1.66	0.10

Visual-Auditory	0.06	0.00	–2.54	**0.02** ^a^

**Significant Clusters**	**ET compared to HC FC**	**t-score**	**cluster contiguous voxels**	

B. Motor Thalamus-Rt. sensorimotor cortex	(+)	≥ 2.9*	113	

B. Motor Thalamus – B. occipitoparietal lobes	(–)	**≥ 4.0** ^a^	8	

B. Motor Thalamus – Cerebellum	(–)	**≥ 2.0** ^a^	1161	


^a^ Significant results.

### Primidone Affects Thalamic-Lateral Somatomotor Network FC

**Figure 3:**
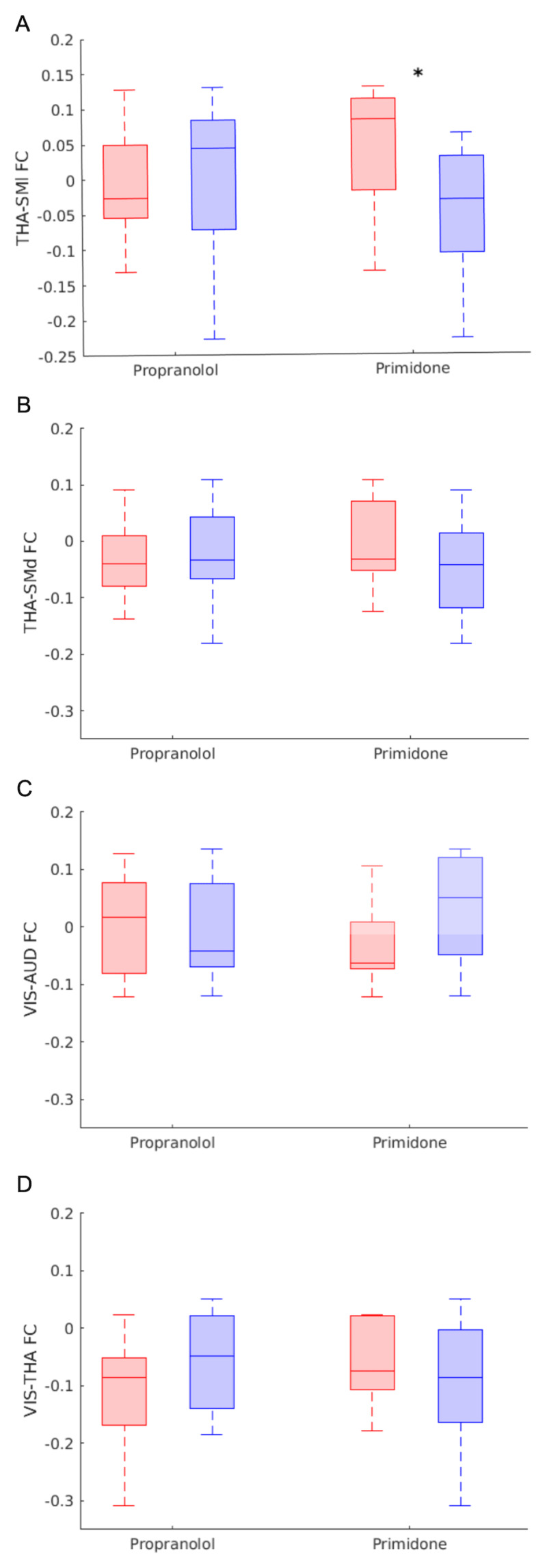
**Medication effects on network FC.** Composite FC scores for (clockwise from top left) thalamus-lateral somatomotor, visual-auditory, thalamus-dorsal somatomotor, and visual-thalamus networks in ET participants taking (red) and not taking (blue) propranolol (*N*: taking = 9, not taking = 12) or primidone (*N*: taking = 10, not taking = 11) at the time of MRI. The central line within each box indicates median FC. The bottom and top edges of the box represent the 25^th^ and 75^th^ percentiles, respectively. The whiskers indicate the minimum and maximum FC values within each group. *P < 0.05, uncorrected.

Primidone use in the ET group was associated with increased thalamus-lateral somatomotor network-level FC (*Mdn:* taking = 0.08, not taking = –0.03, U = 22, p = 0.02), but not thalamus-dorsal somatomotor, thalamus-visual, or visual-auditory FC. Propranolol had no systematic effect on FC in any of the tested networks (Figure 3).

### Altered A Priori Seed-Based Thalamic Regional FC

**Figure 4:**
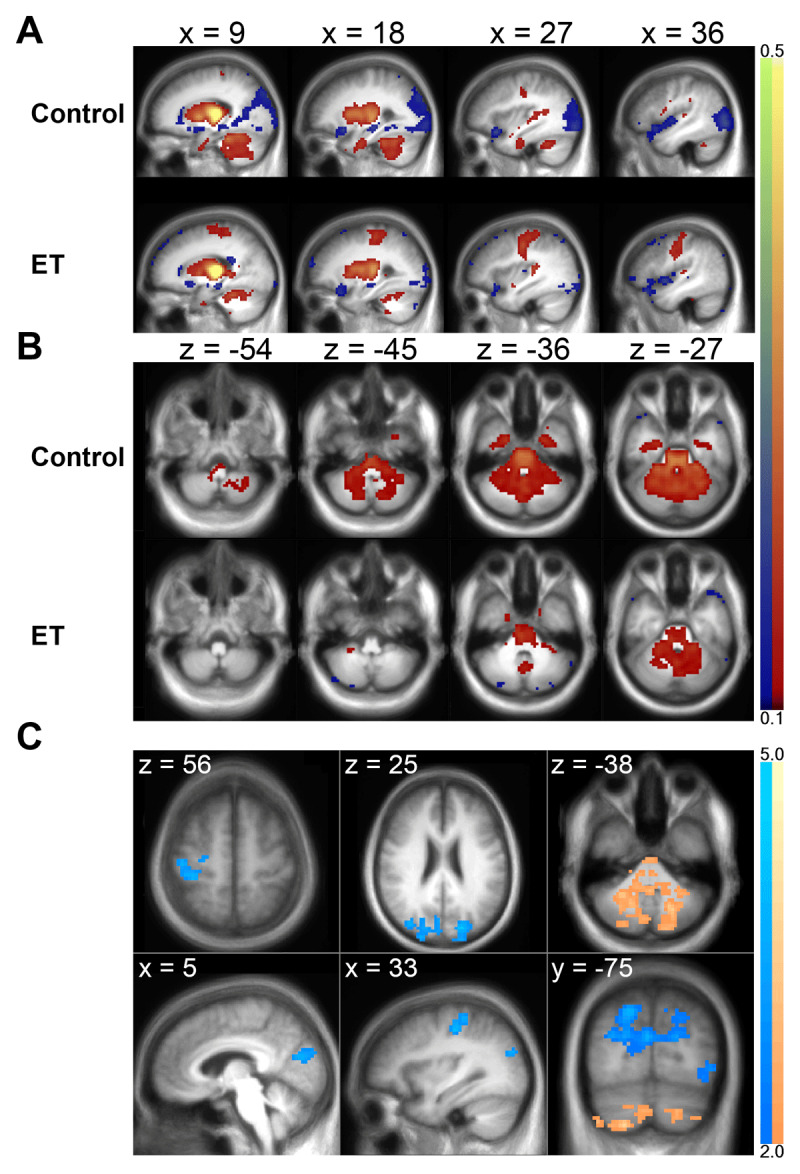
**Bilateral motor thalamus FC in ET and controls. A-B:** ET and control group average correlation maps for a bilateral motor thalamus seed are depicted in the (A) sagittal and (B) axial planes. In both groups, motor thalamus has positive FC with sensorimotor areas, cerebellum, and the whole thalamus and negative FC with occipitoparietal and superior temporal lobes. Color maps are thresholded at |z| ≥ 0.1. Warm colors represent positive and cool colors represent negative correlations. C: Clusters of significant group difference are shown for the group effect z-score subtraction map (control minus ET) in the axial, coronal, and sagittal planes. The ET group has significantly increased FC with right sensorimotor cortex and decreased FC with occipitoparietal lobes, depicted in the first two columns. Note, ET has an increased magnitude of positive FC with sensorimotor cortex and decreased magnitude of negative FC with occipitoparietal cortex, both of which appear as net negative values in the difference map. The cerebellum has decreased FC in ET, shown in the rightmost column.

Both healthy control and ET groups had similar patterns of bilateral motor thalamus seed-based regional FC (Figure 4A-B). ET and healthy control motor thalamus regional FC patterns significantly differed in three clusters (Figure 4C): 1) right sensorimotor cortex, 2) bilateral occipitoparietal lobes, and 3) cerebellum (decreased in ET, highest level of significance t ≥ 2.0). Table 3 summarizes significant seed-cluster results.

### Visual-Lateral Somatomotor Network FC Correlates with Tremor Improvement from DBS

We calculated multiple linear regressions to predict visual-lateral somatomotor FC, thalamus-somatomotor FC, cerebellum-thalamus FC, and thalamus-visual FC based on ΔTRS_OFF-ON_, primidone status, and the MRI scanner on which data were collected. Results of the multiple linear regression indicated that there was a strong collective significant effect between ΔTRS_OFF-ON_, primidone status, MRI scanner, and visual-lateral somatomotor FC, (F(3, 17) = 6.05, p = .005, R2 = 0.52, R2adj = 0.43). The individual predictors were examined further and indicated that ΔTRS_OFF__-ON_ (t = –2.482, p = .024) and primidone status (t = –3.311, p = .004) were significant predictors in the model, and MRI scanner (t = –1.049, p = .309) was a non-significant predictor in the model. There was a moderate collective non-significant effect between ΔTRS_OFF-ON_, primidone status, MRI scanner, and thalamus-somatomotor FC, (F(3, 17) = 2.78, p = .073, R2 = 0.33, R2adj = 0.21). ΔTRS_OFF-ON_, primidone status, and MRI scanner did not predict cerebellum-thalamus FC, (F(3, 17) = 1.02, p = .410, R2 = 0.15, R2adj = 0) nor thalamus-visual FC, (F(3, 17) = 0.69, p = .569, R2 = 0.11, R2adj = –0.05). The regression model did not include propranolol status as it did not have a significant effect on any of the tested networks.

## Discussion

This study demonstrates whole-brain, network, and regional-level FC dysfunction in people with medically refractory ET and identifies visual-lateral somatomotor FC as a potential biomarker to predict tremor improvement after VIM DBS. OODA characterized group differences in the whole-brain correlation matrix without requiring substantial dimensionality reduction. This permitted the unbiased interpretation of FC differences within the larger context of the entire brain correlation matrix followed by focused inspection of functional relationships between regions. The findings of our whole brain analysis support extant literature including altered cerebello-thalamo-cortical FC [47] and highlight the potential relevance of other networks in the pathophysiology of ET. We identified robust differences in visual network FC. Furthermore, tremor improvement after VIM DBS is a significant negative predictor of visual-lateral somatomotor FC.

Abnormal thalamic FC is among the most consistent finding across functional neuroimaging studies of ET [4,48,49,50,51,52,53,54,55]. We demonstrated increased average network-level FC between thalamus and somatomotor networks in ET.

Many neuroimaging studies show cerebellar involvement with ET, although the nature and location of these findings vary [56,57,58]. Electrophysiology and lesion studies provide strong evidence connecting action tremor to the cerebellum [59,60]. Some pathology studies report cerebellar degeneration as evidenced by a loss of Purkinje cells, their connections, and atrophy in ET [61,62], but the role of cerebellar pathological changes in ET is controversial [63].

We did not find abnormal cerebellum FC at the network level in ET, but we did identify decreased regional FC between bilateral motor thalamus and broad regions of the cerebellum. Several possibilities may account for this discrepancy. Our cerebellum network of 27 functionally-defined seeds provides broad coverage of the cerebellum but does not sample areas of potential importance to the pathophysiology of ET such as the deep cerebellar nuclei. Furthermore, we may not have detected a small magnitude network-level difference with our relatively small sample of 21 participants with ET. Finally, regional FC differences do not necessarily translate into altered network-level FC which are highly robust and stable [64]. Thus, differences in the cerebellum resting state networks may not be sensitive to focal cerebellar pathophysiology.

Visual inter-network FC differences featured prominently in our study. Neither primidone nor propranolol related to group-level differences in thalamus-visual FC, suggesting medications did not drive this effect. The role of visual networks in the pathophysiology and therapeutic mechanisms of ET have been increasingly recognized. ET is associated with abnormal visual network FC [47,12,65], where increased visual feedback exacerbates ET’s tremor and relates to fMRI signal changes in cerebello-thalamo-motor, visual, and parietal areas [66]. Interestingly, removal of visual feedback attenuates intention tremor [67]. Although visual cortical regions are not generally recognized as involved in the pathogenesis of ET, our findings add to the growing body of work suggesting that visual-motor and visual-thalamic FC may relate to tremor reduction after thalamic surgeries [18,19,20,21]. Asynchrony between motor and visual systems may explain a visually-responsive action tremor with associated functional abnormalities in motor, subcortical, and visual networks. Discrepancies in visual feedback and motor system timings could plausibly induce periodic and aberrant movement “corrections.” We demonstrate that visual-lateral somatomotor network FC is negatively associated with both tremor improvement after VIM DBS (i.e. weaker FC is associated with a stronger VIM DBS treatment response) and primidone status. This suggests that this network may reflect a shared therapeutic mechanism between different treatment modalities.

Although clinicians characterize ET as a pure motor disorder, patients have subtle impairments compared with age-matched healthy controls in complex visual and auditory attention and visuospatial function [68]. Abnormalities in visual network FC may also reflect these deficits. Prominent differences in visual internetwork FC are also observed in sleep versus awake conditions. In comparison to wake, drowsiness and non-rapid eye movement (NREM) sleep markedly increase the amplitude of spontaneous BOLD activity [69,70], particularly in primary sensory and motor cortices [71,72,73,74,75]. This effect manifests as enhanced resting state functional connectivity (FC) within and between somatomotor and visual areas. Similarly, arousal fluctuations in nominally awake participants cause FC variability to be focally increased in these same areas [76]. Impaired sleep is not an established feature of essential tremor, however persons with essential tremor report higher levels of daytime sleepiness, possibly related to medication side effects [77]. Differences in arousal level may contribute to our findings, however we asked participants about level of wakefulness after each resting-state fMRI run and excluded those in which participants self-reported drowsiness or sleep. The similarities between our data and sleep-wake effects may be spurious given that sleep is grossly normal in essential tremor [78], and we also did not observe group differences in visual-motor FC that are expected with differences in arousal level [79,80]. Furthermore, the magnitude of visual-somatomotor network FC was decreased in ET compared to controls, which is opposite of what would be expected if sedating medications or increased drowsiness were driving FC differences. Alternatively, the similarities may reflect common mechanisms involving the visual sensory network.

Resting-state networks are highly stable and conserved across individuals, arousal states, and even mammalian species [79,80,81]. Our findings of altered whole-brain and network- level FC in ET stand in contrast to similar analyses in focal dystonia in which only regional FC is altered while whole-brain and network-level FC are preserved despite the greater sample size and statistical power in the focal dystonia study [26,27]. These data suggest that in comparison to focal dystonia, abnormalities in ET are more widespread across the brain and/or involve comparatively more network hub regions.

Our study design had several important limitations. First, participants were taking medications at the time of scanning, although these medications were not altered at the time of measured DBS response. Primidone related to increased FC between thalamus and lateral somatomotor networks, however prior work in medication-naïve individuals with ET has also shown increased thalamus-somatomotor FC [58], suggesting medication effects do not fully account for the differences in thalamo-cortical FC observed in this study. Though propranolol did not significantly relate to FC in any of the networks tested, this does not rule out that it may have a modest impact on FC which our study was not powered to detect. Larger studies are necessary to clarify how tremor-suppressing medications impact functional connectivity. Another significant limitation is that DBS outcome measures were limited to the initial programming session when settings are not yet optimized. Unfortunately, subsequent programming sessions in the current cohort did not include OFF TRS assessments. Comparisons between pre-operative TRS and optimized ON TRS at these later sessions were confounded by the effects of time and medication changes (many participants were able to wean medication after DBS). As such, the initial programming OFF vs ON TRS scores were the best proxy of DBS response in our data, though ideally, future studies would assess tremor in OFF vs ON states during follow-up programming sessions. Our study also did not include collection of cognitive performance measures, limiting our ability to assess non-motor correlates of FC. Finally, the relatively small sample size and overrepresentation of males in our study limit its generalizability, particularly given the significant heterogeneity within ET [56].

Nevertheless, the rigorous quality control at each step of preprocessing and analysis and novel statistical methods allow characterization of the group correlation matrix in ET while minimizing confounds and artifacts related to motion. Our whole-brain approach using OODA builds upon published work using seed- and ICA-based approaches to demonstrate abnormal cortical and subcortical FC. Furthermore, we provide evidence for robust effects in visual-somatomotor FC. Overall, we found that ET relates to whole-brain, network, and regional-level FC differences localized within thalamus, somatomotor, and visual regions using an unbiased, whole-brain approach to rs-fMRI analysis and that pre-operative visual-lateral somatomotor FC may predict response to VIM DBS.
